# A Novel Paper-Based Reagentless Dual Functional Soil Test to Instantly Detect Phosphate Infield

**DOI:** 10.3390/s22228803

**Published:** 2022-11-14

**Authors:** Reem Zeitoun, Viacheslav Adamchuk, Asim Biswas

**Affiliations:** 1 School of Environmental Sciences, University of Guelph, 50 Stone Road East, Guelph, ON N1G 2W1, Canada; 2 Department of Bioresource Engineering, McGill University, 21111 Lakeshore Road, Montreal, QC H9X 3V9, Canada

**Keywords:** cyclic voltammetry, Mehlich-3 extractant, molybdate ions, plant-available phosphorus, screen-printed electrodes

## Abstract

Soil tests for plant-available phosphorus (P) are suggested to provide offsite P analysis required to monitor P fertilizer application and reduce P losses to downstream water. However, procedural and cost limitations of current soil phosphate tests have restricted their widespread use and have made them accessible only in laboratories. This study proposes a novel paper-based reagentless electrochemical soil phosphate sensor to extract and detect soil phosphate using an inexpensive and simple approach. In this test, concentrated Mehlich-3 and molybdate ions were impregnated in filter paper, which served as the phosphate extraction and reaction zone, and was followed by electrochemical detection using cyclic voltammetry signals. Soil samples from 22 sampling sites were used to validate this method against inductively coupled plasma optical emission spectroscopy (ICP) soil phosphate tests. Regression and correlation analyses showed a significant relationship between phosphate determinations by ICP and the proposed method, delivering a correlation coefficient, r, of 0.98 and a correlation slope of 1.02. The proposed approach provided a fast, portable, low-cost, accessible, reliable, and single-step test to extract and detect phosphate simultaneously with minimum waste (0.5 mL per sample), which made phosphate characterization possible in the field.

## 1. Introduction

Next to nitrogen (N), phosphorus (P) is the most critical element for plant growth and food production throughout the world [1]. Unlike N, P cannot be supplied through biochemical fixation and must be applied through other sources such as commercial fertilizers, animal manures, and plant residues [1]. In most soils, iron, aluminum, and calcium minerals fix P into forms not available to the plants [1]. To secure plants’ P needs, one of the most common fertilizer practices in many areas is to add large quantities of P fertilizers [1,2]. There remains inefficiency within this practice because the added fertilizers exceed that removed by crops [3]. Research has found that less than 15% of fertilizer-applied phosphorus is uptaken by the crop during the year the fertilizer was added [1]. Unregulated and excessive application of P fertilizers results in P leaching into groundwater or entering water streams via agriculture runoff. This has resulted in serious water problems through P build-up in water bodies and agricultural watersheds [4]. High P and N levels are the main causes of eutrophication of streams and lakes where algae grow to a certain extent, causing depletion of the dissolved oxygen in water and sunlight blockage, which lead to the death of fish and macrophytes [5]. Therefore, it is highly recommended to apply P fertilizers to soil on an as-needed basis to preserve the safety of water streams and the overall ecosystem. This has contributed to the development of reliable P detection methods that are fundamental in judging soil P fertility [6,7]. 

**Figure 1:**
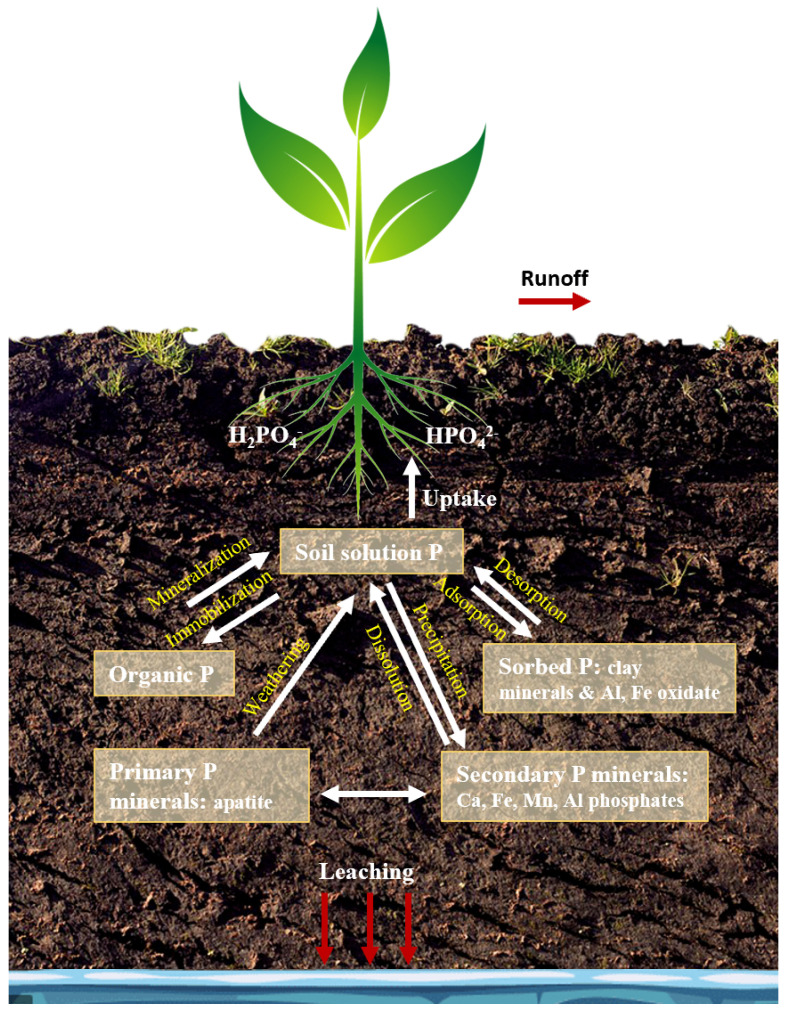
P cycle in soil.

P in the soil can be classified as labile P, which is available to plants or organisms, and non-labile P, which is stable and exists in insoluble forms [8]. Labile P comes from different soil P pools in the soil. Labile P can be adsorbed on clay minerals and oxides of iron (Fe) and aluminum (Al) (Figure 1), or it can be the mineralized fraction of organic P which is typically mineralized after a short time. The non-labile P fraction in soil includes the stable organic form that can remain in this form for years and the precipitated forms of inorganic P such as Al, Fe, manganese (Mn), and calcium (Ca) phosphates (Figure 1). The modern soil P tests provide an indication of the labile soil P available to the plants, P in soil solution, and non-labile P which becomes available slowly, but not the total concentration of P.

Spectroscopy in the visible and near-infrared (Vis–NIR) and in the mid-infrared (MIR) has been used in previous works to elucidate relationships between soil spectra and PAP status. The mobile and non-invasive nature of spectrometers makes them very attractive in soil science tests; however, the practical applicability of PAP prediction, using Vis–NIR or MIR, in precision agriculture is not yet satisfactory for the determination of precision fertilizers dosage. Reviews by Kuang et al. (2012) and Soriano-Disla et al. (2014) have reported that IR spectroscopy provides only an approximate quantitative prediction of PAP mainly because of the low dipole moment between P and oxygen which inhibits the detection of orthophosphates [9,10].

Traditionally, soil scientists depend on soil test P values to guide P fertilizer application [11]. A viable soil P test requires the development of two phases of research: a correlation phase and a calibration phase. The correlation between P extracted by chemical extractant and the P amount available to the plants is necessary towards selecting the right chemical extractant [12]. PAP is extracted from soil samples using different chemical extractants that require lengthy extraction time, agitation, and sample filtration [13] and is subsequently detected using inductively coupled plasma optical emission spectroscopy (ICP) and colorimetric tools [14,15,16,17] to assess soil P needs. However, these detection tools are accompanied by many challenges such as extensive maintenance, high annual consumption costs, complicated steps, requirement of trained personnel, immobility, and lengthy analysis time [14,15,16,17]. Mehlich-3 [18] and Olsen [19] are two chemical extractants that are widely used in soil analysis laboratories to extract labile PAP and have consistently shown a good correlation with P uptaken by plants [20,21,22]. Furthermore, Mehlich-3 serves as an extractant for several nutrients such as magnesium (Mg), sodium (Na), Ca, potassium (K), copper (Cu), zinc (Zn), and Fe. Mehlich-3 extractant has five components with each having different functions towards dealing with the Ca-Fe-Al-P complex. The fluoride ion is the primary component of Mehlich-3 for P selective extractability. The fluoride ion dissolves the aluminum and iron-bound phosphate, releasing P and forming Al-F and Fe-F complexes [18,23,24]. In addition, the ammonium ion facilitates in extracting basic cations such as magnesium, sodium, calcium, and potassium [23]. Ammonium nitrate reacts with acetic acid to form ammonium acetate, and its ammonium ion complements the ammonium fluoride in extracting basic cations [18,23]. Nitric acid extracts a portion of calcium phosphates, and its acid components aid in the extraction of basic and micronutrients cations [18,23]. Ethylenediaminetetraacetic acid (EDTA) and ammonium nitrate form complexes with copper, zinc, iron, and manganese to release phosphate from heavy metal phosphates [25,26]. EDTA is also accountable for preventing precipitation of calcium fluoride [18,23]. Acetic acid is used to maintain a pH less than 2.9 to prevent calcium from precipitating as calcium fluoride [18,23]. Acetic acid also serves in decomposing apatite and thus releasing P [18]. Olsen P extractant is typically used in alkaline and neutral soils and has only one component, sodium bicarbonate, which enhances the dissolution of calcium–phosphate through the precipitation of calcium carbonate. 

In North America, Mehlich-3 is typically used in areas with acidic to neutral soils because the free lime in soil can neutralize the acid, which can underestimate the available phosphorus in soil [20]. However, several studies have shown high linear correlation (r = 0.93 [24], r = 0.81 [21], r = 0.99 [27]) between PAP extracted by Olsen and Mehlich-3, suggesting comparable results between Olsen and Melich-3 extractable P in calcareous soils. In addition, conversion equations have been suggested to allow for the combination of data derived by Olsen and Mehlich-3 extraction methods [28]. The calibration phase of the soil P test involves determining the crop nutrient requirement at soil test values to produce quality responses to the added fertilizers. The calibration phase has been developed for Mehlich-3 and Olsen soil P tests in previous studies for different crop production [20,22]. In this proposed method, Mehlich-3 components, along with P detection reagents, were impregnated in filter papers to extract P due to its high selectivity towards P extraction and its ability to extract P in a short period [18] (5 min extraction) as opposed to other P extractants which require longer extraction times. In our previous study, our team developed an electrochemical method for inorganic soil P determination in Olsen P extractants based on anodic oxidation of phosphomolybdate [14,29]. However, this method could not be deployed in infield P detection because it requires safe disposal of the extractant solution chemical wastes. The aim of the present work is to expand our method towards a reagentless dual functioning assay that can be used in onsite P testing as a standalone sensor to extract and detect soil P simultaneously. In this study, we integrated, for the first time, an efficient reagent-free P extraction and detection analyses on a single piece of impregnated filter paper. Subsequently, the filter paper was characterized for its surface crystallization morphology, chemical-releasing capacity, and time required for maximum chemical recovery. The proposed assay has been electrochemically characterized by implementing cyclic voltammetry (CV) to detect cathodic peak current. The easy fabrication, portability, chemical-free, and fast responses of the proposed method make it useful for infield soil P tests and accessible to not only agronomists but also farmers and land growers.

## 2. Materials and Methods

### 2.1. Extraction–Reaction Reagent, P Standards, and Interfering Solutions

An extraction–reaction reagent (ERR) was prepared to extract plant-available phosphorus (PAP) and react with molybdate-reactive P (inorganic soil phosphate) concurrently. ERR was prepared by mixing acidic molybdate (AMT) ions with concentrated Mehlich-3 solution (×5). Using a calibrated 100 mL flask, the chemicals of concentrated Mehlich-3 were first added to the flask by thoroughly dissolving 50 g of ammonium nitrate (NH_4_NO_3_), 1.39 g of ammonium fluoride (NH_4_F), 0.73 g of ethylenediaminetetraacetic acid (EDTA) in 50 mL of deionized (DI) water. Following this, 28.75 mL of acetic acid (CH_3_COOH) and 2.05 mL of nitric acid (HNO_3_) were added to the flask and mixed thoroughly. Then, 4.63 g of ammonium molybdate tetrahydrate ((NH_4_)_6_Mo_7_O_24_ · 4H_2_O) and 3.80 mL of sulfuric acid (H_3_SO_4_) were added to the concentrated Mehlich-3 mixture to bring down the pH to 0.35. DI water was added to bring the total volume to 100 mL. P standard solutions were prepared by dissolving 136 mg of potassium dihydrogen phosphate (KH_2_PO_4_) in 1 L of DI water to prepare the stock solution, which was diluted to make 0.15–10 mg·L^−1^ (5.00 × 10^−6^–3.23 × 10^−4^ M) P standard solutions. To evaluate the interference of silicate ions with phosphate electrochemical detection, 4.70 mg of sodium hexafluorosilicate (F_6_Na_2_Si) was dissolved in 100 mL of DI water and 100 mL of 0.15 and 7.74 mg·L^−1^ P standard solutions. All chemicals were purchased from Fisher Scientific, Mississauga, ON, Canada.

### 2.2. Impregnated Filters Preparation 

Different filters were tested in this study to determine the effect of pore size and filter thickness on reagents recovery. Whatman 41, 42, 934-AH (purchased from Fisher Scientific, Mississauga, ON, Canada, and Millipore AP25 124 50 (purchased from MilliporeSigma, Oakville, ON, Canada) filters were cut into 0.5″ × 0.5″ squares using EK Success Tools Square punch and spread in Petri dishes. To prepare the impregnated filter paper (IFP), 100 µL of ERR was drop-casted, using a pipette, on each filter square and were left overnight to dry. 

### 2.3. Physical Characterization of ERR IFP

The surface morphology of the impregnated filters was characterized using USB Digital Microscope 40× to 1000×.

### 2.4. Recovery of Reagents from ERR IFP

Impregnated Whatman 41, 42, and 934-AH and Millipore AP25 124 50 filters were tested for recovery or release of reagents. Five impregnated filters of each were suspended in 2.5 mL of DI water for 10 min, and the conductivity of the suspended solution was detected using Fisherbrand™ accumet™ XL600 Dual 144 Benchtop meter. The filter showing the highest recovery was used in the rest of this study. To determine the time needed to completely release the impregnated reagents from the filters, the release of nutrients from the impregnated filters was tested over different time intervals (0, 30, 60, 90, 120, 180, 240, 300, 360, 420, 480, 540, and 600 s).

### 2.5. Stability of ERR IFP

Whatman 41 0.5″ × 0.5″ filters were impregnated with 100 µL, for each square, of two reagents to evaluate and compare the loss of chemicals through evaporation of volatile components such as acetic and nitric acid. The two reagents used are ERR and ERR without acetic and nitric acids (ERR w/o AA + NA). The recovery of the two reagents from the filters was tested by measuring the conductivity of filters suspension over one-week using Fisherbrand™ accumet™ XL600 Dual 144 Benchtop meter. 

### 2.6. Soil P Extraction 

**Figure 2:**
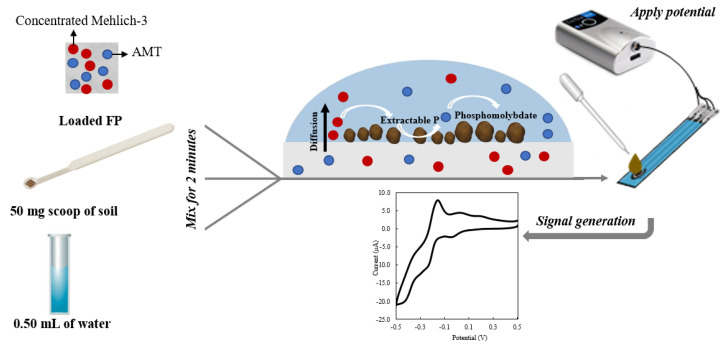
Setup scheme for soil phosphate analysis using ERR IFP.

A 50 mg capacity soil test measuring spoon (Lamotte Chemical, Chestertown, MD, USA) was used to weigh 50 mg of soil and mix it with 0.50 mL of DI water and 1 ERR IFP for 1, 2, 5, and 10 min (Figure 2). The suspended solution was used to quantify P concentration via cyclic voltammetry (CV) (Figure 2); the CV settings used are found in Section 2.9. The extraction or mixing time showing the least difference between the CV and ICP Mehlich-3 results was applied to the rest of the soil samples.

### 2.7. Soil Sample Collection and Laboratory Analysis

**Figure 3:**
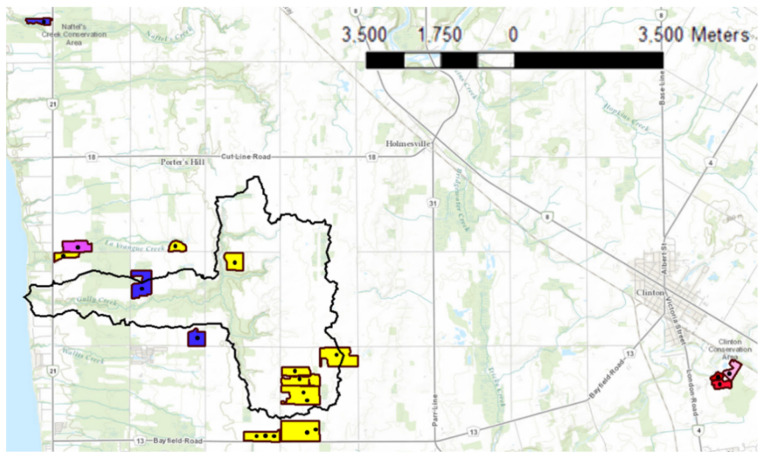
The locations of soil sampling points on the soil map and the soil texture of the fields. Legend: ▬ Gully Creek boundaries, ▬ field sites boundaries, ■ clay loam, ■ loam, ■ silty clay ■ sandy loam, ■ silty clay loam.

Twenty-two soil samples were supplied to our laboratory by Agriculture and Agri-Food Canada (AAFC). Figure 3 represents the locations and soil types of the sampling points within the Gully Creek watershed (~15 km^2^) located in the southwest of Ontario (Canada) in Huron county and outside of the watershed boundaries (e.g., Huronview field). Seventy percent of the watershed is covered by cropland, and the remaining areas consist of shrubs, forests, and meadows. Winter wheat, corn, and soybean are the main crops grown in the watershed [30]. A minimum of 25 soil cores (0–15 cm depth) were randomly collected using a soil core sampler for each agricultural field (<20 acres). The soil cores were mixed in a bucket, producing one composite sample per field. The soil samples were stored in Ziploc plastic bags and transported to our lab at the University of Guelph for analysis. Prior to Mehlich-3 extraction, the 22 soil samples were air-dried and sieved to <2 mm. PAP was extracted using the traditional extraction method, Mehlich-3. The concentration of P in the extracts was determined using inductively coupled plasma optical emission spectroscopy (ICP) (model: 174 Varian VISTA PRO, CCD Simultaneous Axial ICP-OES). Due to the high cost of ICP-OES tests, two soil samples were collected from each soil composite, extracted separately, and tested for PAP. Soil pH was measured in a 1:2 soil/DI water suspension using a pH meter. The average, maximum and minimum soil pH were 7.58 ± 0.03, 7.76, 7.20, respectively. To determine the total concentration of aluminum, iron, and calcium, the soil samples were digested in a 3:1 mixture of hydrochloric (HCl) and nitric (HNO_3_) acids following the modified microwave acid digestion method (Method 3051a US EPA 2007). The total aluminum, iron, and calcium ranges of the 22 soil samples were 6200–23,222, 6700–23,000, and 2496–67,425 mg·kg, respectively.

### 2.8. Soil P Data Mapping

Soil P data collected from the proposed method and ICP analysis were used to generate a map showing P fertilizer recommendation based on the soil test value. This was generated by ArcGIS 10.8.1. P soil test index values were adapted from Mallarino et al., 2013 [22]. The interpretation of these soil-test values was determined using recommended analysis methods (6-inch-deep soil samples) and handling procedures. Soil-test values for each P method were classified into interpretive categories which included very low, low, optimum, high, and very high.

### 2.9. CV Electrochemical P Detection

CV scans of P tests were conducted by EmStatBlue potentiostat (manufactured by PalmSens—Compact Electrochemical Interfaces; Houten, The Netherlands) connected via Bluetooth to Microsoft surface pro tablet (Surface Pro 6 Model 1796 i5; Microsoft, Redmond, WA, USA). Zensor TE100 screen-printed electrodes (SPE) were obtained from eDAQ Pty Ltd. (Colorado Springs, CO, USA), modified following our previous work [29], and connected to the potentiostat via alligator clips. For each P standard solution, one ERR IFP was suspended in 0.5 mL of P standard for two minutes. Disposal pipettes were used to dispense the suspended solution on the SPE. CV scans were obtained between −0.5 and 0.5 V under a scanning rate of 50 mV·s^−1^. PSTrace software was used to generate the differintegral view of the cathodic sweep of each CV scan and to locate the potential of the second reduction peak. This potential was used in the CV to quantify the peak height of the second reduction peak and correlate it with phosphate concentrations to generate the calibration curve. 

### 2.10. Data Analysis

Statistical analysis was performed using Microsoft Office 365 Excel. All results were quadrupled and expressed as mean ± standard error ($\sigma_{\overset{-}{x}}$). SAS^®^ software package (SASonDemand, SAS Institute Inc., Cary, NC, USA) was used to conduct Tukey’s honest significance difference to compare the mean values of different measurements, with *p* < 0.05 indicating a significant difference. The fit performance of the calibration curve was expressed using the coefficient of determination (R^2^). Regression analysis was also assessed to estimate the correlation between ICP Melich-3 P and CV P (using ERR IFP). 

## 3. Results and Discussion

### 3.1. Chemical and Physical Characterization of Extraction–Reaction Reagent Impregnated Filter Paper 

Whatman 41 filter showed the highest recovery of released chemicals having a value of 59.47 ± 0.24 % (Table 1). Hence, it was used in the soil P tests in this study. It was observed that the recovery of released chemicals was affected by two factors, the filter paper (FP) thickness, and pore size. The very high thickness of Millipore AP2512450 led to minimal recovery of chemicals of 0.21 ± 0.02%. The thicker the FP, the more liquid it can retain, leading to an absorbance of the water added to the soil sample to test for extractable P and less chemicals being released to the water compared to thinner FP’s (Table 1). Smaller pore size could result in restricted counter diffusion of the solvent to the bulk solution, resulting in lower recoveries (Table 1). The same effect of pore size was observed on a drug release study performed on mesoporous silica nanoparticles [31]. 

**Figure 4:**
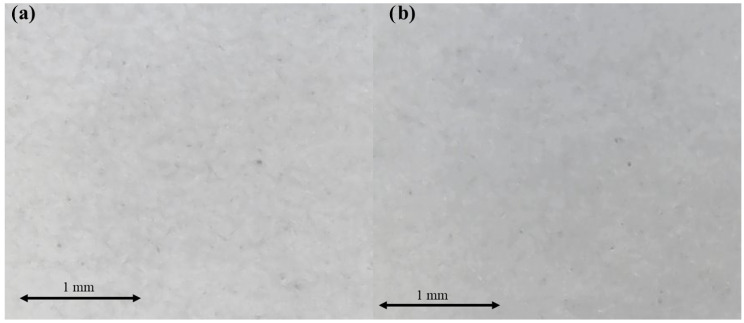
Digital microscope images of (**a**) unmodified Whatman 41 FP and (**b**) impregnated Whatman 41 FP.

Unmodified and impregnated Whatman 41 FP’s showed no morphological difference (Figure 4). No surface crystallization was observed on the impregnated Whatman 41 FP (Figure 4b). This indicated that the solvent was loaded within the pores of the FP, and these impregnated FPs could be handled safely by any personnel. 

Component volatility of NA and AA was studied to assess the effect of the individual element volatility on chemical losses from extraction–reaction reagents impregnated in filter paper (ERR IFP). The recovery of chemicals was almost the same in ERR IFP and ERR IFP without acetic and nitric acids (AA + NA) throughout the six days (Table 2). This indicated that the chemical losses were not associated with NA and AA volatility losses. Hence, it can be assumed that the ERR mixture is an ideal solution, and no interactions were taking place in the mixture. The ERR solution consisted of three solvents: water, AA, and NA. Raoult’s law (Equation (1)) was used to calculate the vapor pressure of the mixture to be equal to 17.77 mmHg (20 °C).

(1) $$P=\overset{N}{\underset{i=1}{\sum }}x_{i}P_{i}^{sat}$$

where *x_i_* is the liquid phase mole fraction of component *i*, and *P_i_^sat^* is the vapor pressure of component *i*. The vapor pressure of the mixture was almost equal to the pure water vapor pressure of 17.53 mmHg (20 °C).

**Figure 5:**
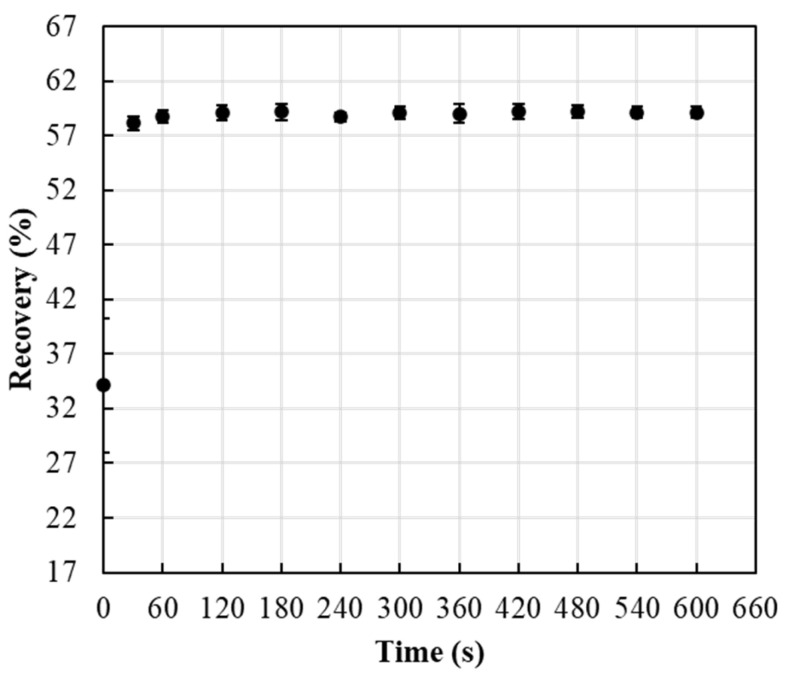
Effect of time on ERR release from ERR IFP.

ERR released from IFP showed an immediate release after 60 s (Figure 5). The release process of the ERR reagent molecules involves the reagent dissolving in the dissolution medium and escaping from the FP pore channels. Therefore, the large FP pores allow the dissolution medium to penetrate promptly into the carriers’ channels, and the reagent molecules will have a higher chance of being released immediately. 

### 3.2. CV Response Characteristics

**Figure 6:**
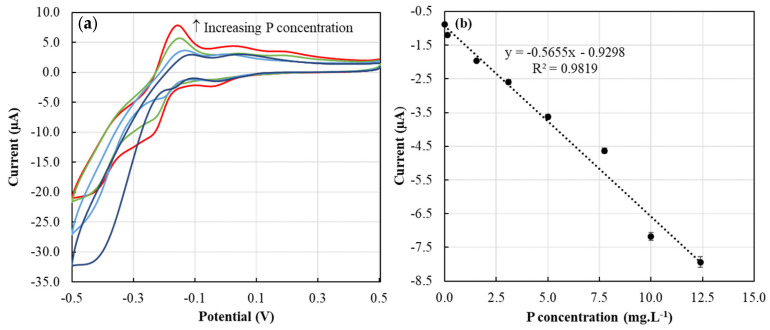
(**a**) Cyclic voltammograms of 1 ERR IFP suspended in 0.5 mL of ▬ 10 mg·L^−1^, ▬ 7.74 mg·L^−1^, ▬ 3.10 mg·L^−1^, and ▬ 0.15 mg·L^−1^ of P standards for two minutes; (**b**) calibration curve corresponding to P standard solutions.

The CV approach used in this study was based on our previous work [14]. The phosphate ion in the dissolution medium reacted with acidic molybdate (AMT) released from the ERR IFP, forming a complex Keggin phosphomolybdate anion [14,32]. The observed anodic and cathodic peaks (Figure 6a) are due to the two steps of reduction reaction (Equations (2) and (3)) and corresponding oxidation reactions [14,33].

(2) $$7PO_{4}^{3-}+12Mo_{7}O_{24}^{6-}+72H^{+}\to 7PMo_{12}O_{40}^{3-}+36H_{2}O\;$$

(3) $$7PMo_{12}O_{40}^{3-}+ne^{-}+nH^{+}\to {\left[H_{n}PMo_{12}O_{40}\right]}^{3-}$$

The calibration curve showed a high sensitivity response towards P sensing, exhibiting a slope value of −0.56 µA/(mg·L^−1^). The second cathodic peak chosen for this work was found to show a good linear relationship with P concentration (Figure 6b), showing a high coefficient of determination (R^2^) value of 0.98. 

### 3.3. Silicate Interference

**Figure 7:**
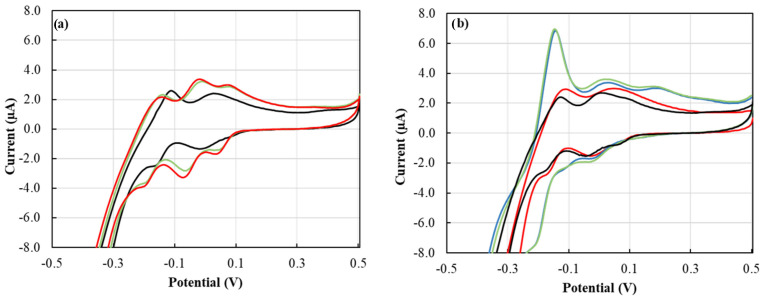
(**a**) Cyclic voltammograms of 1 ERR IFP suspended in 0.5 mL 7.74 mg·L^−1^ Si for ▬ 2, ▬ 5, and ▬ 10 min; (**b**) cyclic voltammograms of 1 ERR IFP suspended in 0.5 mL of 7.74 mg·L^−1^ P▬, 0.15 mg·L^−1^ P ▬ 7.74 mg·L^−1^ P and 7.74 mg·L^−1^ Si ▬, and 0.15 mg·L^−1^ P and 7.74 mg·L^−1^ Si ▬, for 2 min.

From our previous work, our proposed cyclic voltammetry (CV) P tests showed excellent selectivity towards P with no interference from several nutrients that could be found in the soil, such as nitrate, chloride, and sulfate [14]. The concentration of soluble silica range from 1 to 40 mg·L^−1^ in soil with a value of 15 to 20 mg·L^−1^ is most commonly found in neutral and acidic soils, where silica solubility is limited by amorphous silica [34]. Dissolved silica in soil samples could create a cross-interference with phosphate in the CV tests. Silicate interference with the phosphomolybdate complex formation can be avoided by addressing the protons-to-molybdate ratio [35] and the reaction time [34,36]. Silicate interference could be avoided by allowing the protons-to-molybdate ratio to be close to 70 [35]. This was achieved by adjusting the pH of the reagent to pH 0.35 using sulfuric acid and providing a molybdate molar concentration of 7.5 mM. The CV signal of the silica medium mixed with ERR IFP for two minutes delivered a peak height of −1.50 µA which corresponded to a P concentration of 0 mg·L^−1^ (Figure 7a). This showed that silica would not interfere with P detection at two minutes reaction time, and thus, two minutes were used as a reaction time throughout this work. Furthermore, the Si/P ratio of 1 and 51.6 (Figure 7b) did not show any difference in the peak height from only P medium signals. However, after five minutes of reaction time, the silica showed unique voltammetry from P voltammetry (Figure 7a) where the waves overlapped to a greater extent, were less steep, and shifted towards negative potential. Similar behavior was observed in silicomolybdate complex voltammetry [37]. 

### 3.4. Soil Samples Analysis

An extraction time of 1 min showed significantly lower recovery of soil P (Table 3) compared to 2–10 min extraction. In addition, 2–10 min of extraction time was required to deliver higher recoveries (Table 3) and is more representative of P extracted by traditional Mehlich-3 soil P test. In this study, 2 min extraction was used throughout the tests. A longer extraction time could lead to AMT reaction with interfering silicate, as indicated in the previous section and hence could overestimate P. 

Extracting soil PAP is a complicated procedure that typically involves four steps: 1—preparing P extractant solution (Table 4), 2—mixing soil sample with extractant at specific weight/volume ratio (Table 4), 3—agitating the sample for a length of time (Table 4), and 4—filtering the soil/extractant mixture. This takes time and effort and needs proper disposal of chemical wastes. In addition, this is not suitable for rapid PAP quantification. The proposed method is useful for rapid PAP detection because it requires less extraction time (2 min) than other conventional PAP extractants (Table 4), eliminating the need to agitate and filter soil/extractant mixture. 

**Figure 8:**
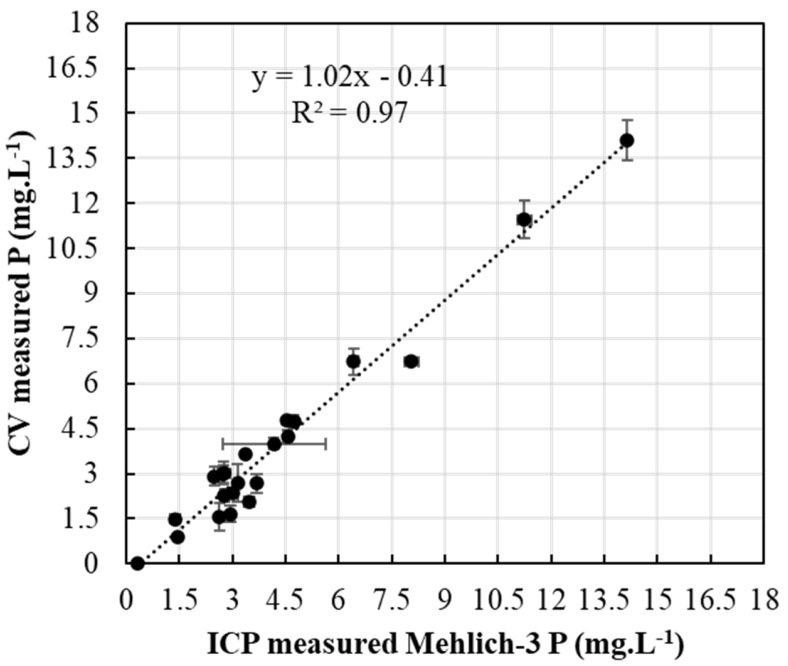
Measured CV (using ERR IFP) soil P versus ICP Mehlich-3 soil P.

PAP extracted and analyzed with ERR IFP CV setup (Figure 2) was compared to Mehlich-3-extracted PAP analyzed with ICP (Figure 8) for the same soil samples. Regression and correlation analyses showed a highly significant relationship between P determinations by the ICP Mehlich-3 and ERR IFP CV P, delivering an R^2^ value of 0.97. This shows great potential for the ERR IFP CV approach in soil tests. The CV-detected soil P was almost the same for the ICP soil P as indicated by the correlation slope of 1.02 (Figure 8). The proposed electroanalytical protocol provided a limit of detection of 0.11 mg·L^−1^, which is shown to be effectively applied to alkaline soil samples and which was validated by ICP tests for quantification of PAP in soil Mehlich-3 extracts. Our proposed approach provides a simple and single step protocol to extract and detect PAP simultaneously with minimum waste (0.5 mL per sample), which makes PAP characterization possible in the field.

### 3.5. Soil P Test Interpretation

**Figure 9:**
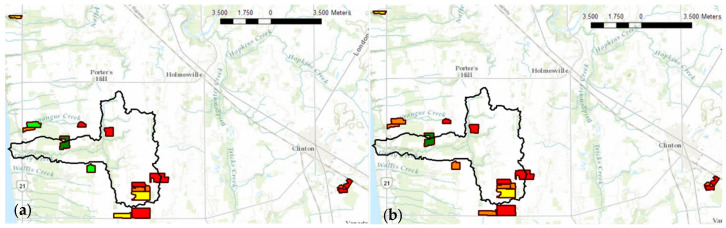
Measured soil-extractable P concentration with regard to crop response to P inputs using (**a**) ERR IFP CV P test; (**b**) Mehlich-3 ICP P test. Legend: ■ too high, ■ high, ■ optimum, ■ low, ■ very low.

The variability of P across the 16 agricultural fields (Figure 9) promotes applying P fertilizers in soil on an as-needed basis to maintain the safety of watersheds and save fertilizer resources. This emphasizes the importance of P analytical development of fast and precise P-detecting methods towards judging soil health and productivity. The soil P recommendations based on ERR IFP CV P test (Figure 9a) were lower than ICP Mehlich-3 (Figure 9b) recommendations for three fields, indicating that ERR IFP CV recommendations were underestimated for these fields. However, this could be due to the presence of organic P in Mehlich-3 extractants, which can be detected by ICP determination but not by CV determination [16]. 

## 4. Conclusions

Conventional accredited detection methods of soil PAP fertility deliver precise measurements for a limited number of samples due to time, cost, and labor involved with laboratory analysis. This makes them insufficient to characterize phosphorus variability within agricultural landscapes and accessible to only specific personnel. Until now, no infield extraction protocol has been developed for P nutrients, requiring the transport of soil samples to the laboratory to extract P. In this study, the extraction and determination of PAP using ERR IFP and CV setup were carried out for the first time. The proposed procedure utilized fast and cost-effective means to extract PAP using ERR IFP. This extraction method required less time (2 min) than other conventional PAP extractants and was comparable to P extracted with Mehlich-3 extractant solution. In addition, less waste was associated with this procedure, requiring only a couple of drops (~0.5 mL) of extraction solution (DI water) to run CV scans. The simple P extraction, portability of setup, zero chemicals to be handled by end user, and CV instant response make this protocol a potential method for infield soil P testing and accessible to any personnel. The spatial coverage and available budget affect the number of soil P tests collected in the agricultural field. With the cost-effectiveness and high accessibility of the proposed method, denser P sampling and testing could take place over the fields. With more soil P data available, continuous P maps with higher resolution can be prepared which will guide more sustainable P management. Research is still required for further standardization of the procedure. Fresh soil samples are wet, and soil water content could affect the concentration of P detected. Eliminating the error introduced by soil water content is under investigation in our future research. 

## Figures and Tables

**Table 1:** Properties of filters used in this study and their observed recoveries of chemicals after impregnation.

Filter Name	Filter Material	Uses	Thickness (µm)	Pore Size (µm)	Basis Weight (g/m^2^)	Recovery (%) ± $\sigma_{\overset{-}{x}}$
Whatman 41	Cellulose	Fast quantitative air pollution analysis as a paper tape for impregnation	220	20–25	85	59.47 ± 0.24 ^a^
Whatman 42	Cellulose	Slow quantitative analysis for filtering extremely small particles	200	2.5	100	53.13 ± 0.25 ^b^
Whatman 934-AH	Binder-free glass microfiber	Fast and high loading capacity filtration	435	1.5	64	51.17 ± 0.69 ^c^
Millipore AP2512450	Hydrophilic glass fiber with binder resin	Prefiltration for heavily contaminated liquids	1200	2	NA	0.21 ± 0.02 ^d^

^a–d^ Letters (a–d) specify statistically significant differences (*p* < 0.05) within each column using Tukey’s honest significance of difference test.

**Table 2:** Recovery of chemicals from ERR w/o AA + NA IFP and ERR IFP after 1–6 days of IFP curing/drying.

	Recovery (%) ± $\sigma_{\overset{-}{x}}$	
Day	ERR w/o AA + NA IFP	ERR IFP
1	60.62 ± 0.80 ^a^	59.44 ± 1.26 ^a^
2	59.55 ± 1.20 ^a^	60.43 ± 0.78 ^a^
3	57.03 ± 2.01 ^a^	58.62 ± 0.56 ^a^
6	57.19 ± 1.34 ^a^	58.71 ± 0.07 ^a^

^a^ represents statistically nonsignificant difference (*p* > 0.05) between days within each column, determined using Tukey’s honest significance of difference test.

**Table 3:** Effect of extraction time on extracted soil P using CV of 1 FP suspended in a mixture of 0.5 mL of water and 50 mg of soil.

Extraction Time (min)	Predicted P Concentration (mg·L^−1^)	Recovery (%)
1	1.75 ± 0.15	42.02 ± 3.68 ^b^
2	4.35 ± 0.23	104.31 ± 4.75 ^a^
5	4.39 ± 0.29	105.23 ± 6.15 ^a^
10	4.43 ± 0.18	106.22 ± 3.79 ^a^

^a,b^ specify statistically significant difference (*p* < 0.05) within each column using Tukey’s honest significance of difference test.

**Table 4:** Overview of soil P extraction methods.

Method	Extracting Solution	Solution pH	Extraction Time	Soil-to-Solution Ratio	Method of Detection	Ref.
HCl	0.5 M HCl	<1	2 h	1:10	ICP	[13,38]
LiCl	0.4 M LiCl	unbuffered	2 × 2 h	1:1.8–1:4.0	ICP	[13]
CAE *	Distilled water	unbuffered	16 h	1:40	photometer	[13]
CAL *	0.05 M C_6_H_10_CaO_6_, 0.05 M (CH_3_COO)_2_Ca	4.0	2 h	1:20	photometer	[13]
CaCl_2_	0.01 M CaCl_2_	unbuffered	2 h	1:10	photometer	[13,39]
H_2_O	Distilled water	unbuffered	12 h	1:0.3–1:1.2	photometer	[13]
FeO IFP *	0.01 M CaCl_2_	unbuffered	16 h	1:40	photometer	[13,40]
Olsen	0.5 M NaHCO_3_	8.5	30 min	1:20	Photometer/ICP	[13,19]
Mehlich-3	0.015 M NH_4_F, 0.013 M HNO_3_, 0.001 M EDTA, 0.25 M NH_4_NO_3_ 0.3 M CH_3_COOH	2.5	5 min	1:10	Photometer/ICP	[13,18]
ERR IFP *	DI water	Unbuffered	2 min	1:10	Cyclic voltammetry	This work

* CAE: cation/anion exchange membranes; CAL: calcium acetate lactate; Fe IFP: iron oxide impregnated filter paper; ERR IFP: extraction–detection reagent impregnated in filter paper.

## Data Availability

The data supporting the findings of this study are available from the corresponding authors upon request.
